# Postprocedural Contrast-Associated Acute Kidney Injury and Prognosis of Patients Undergoing Recanalization of Chronic Total Occlusions

**DOI:** 10.3390/jcm13247676

**Published:** 2024-12-16

**Authors:** Kevin Hamzaraj, Caglayan Demirel, Mariann Gyöngyösi, Philipp E. Bartko, Christian Hengstenberg, Bernhard Frey, Rayyan Hemetsberger

**Affiliations:** Department of Internal Medicine II, Division of Cardiology, Medical University of Vienna, 1090 Vienna, Austria; kevin.hamzaraj@meduniwien.ac.at (K.H.); caglayan.demirel@meduniwien.ac.at (C.D.); mariann.gyongyosi@meduniwien.ac.at (M.G.); philipp-emanuel.bartko@meduniwien.ac.at (P.E.B.); christian.hengstenberg@meduniwien.ac.at (C.H.); rayyan.hemetsberger@meduniwien.ac.at (R.H.)

**Keywords:** chronic total occlusion, contrast-associated acute kidney injury, percutaneous coronary intervention

## Abstract

**Introduction:** Percutaneous coronary intervention (PCI) for chronic total occlusions (CTOs) requires advanced techniques and prolonged procedural efforts, often necessitating high contrast volumes, which may increase the risk of contrast-associated acute kidney injury (CA-AKI). However, evidence suggests that factors beyond contrast exposure contribute to CA-AKI, though data specific to CTO PCI remain limited. **Methods:** Patients undergoing contemporary CTO PCI at our university-affiliated tertiary care center were enrolled. CA-AKI was defined according to KDIGO criteria, and patients were stratified based on the presence of postprocedural CA-AKI. Baseline and procedural characteristics, including osmotic factors, were compared between the groups. The primary outcome was all-cause mortality at one year, and the secondary outcome was all-cause mortality at three years. **Results:** A total of 145 patients were enrolled, with a mean age of 67 years, and 75% were male. Baseline creatinine levels, electrolytes, and osmotic factors did not differ significantly between groups. Lesion parameters and J-CTO scores were also comparable. The contrast volume and procedural duration were numerically higher in patients who developed CA-AKI. Patients with CA-AKI received a higher radiation dose (22.1 vs. 13.2 Gy·cm^2^, *p* = 0.041). CA-AKI emerged as an independent predictor of all-cause mortality at one year (adjusted HR 5.3, CI [1.52–18.51], *p* = 0.009) but not at three years. **Conclusions:** In this retrospective analysis, CA-AKI was an independent predictor of all-cause mortality at one year following CTO PCI but lost predictive value at three years. Baseline renal function and contrast volume alone did not predict CA-AKI. Instead, procedural complexity, reflected by higher radiation exposure, was associated with an elevated risk of CA-AKI.

## 1. Introduction

Historically, acute kidney injury following coronary interventions was primarily attributed to the volume of contrast media administered during angiography and percutaneous coronary interventions (PCI) to guide the procedure [1]. However, recent definitions have shifted the focus away from contrast volumes alone, raising whether other factors, such as embolization or hemodynamic impairment during the procedure, equally contribute to procedure-related acute kidney injury [1]. As a result, the term “contrast-induced nephropathy” (CIN) has been replaced by “contrast-associated acute kidney injury” (CA-AKI). Despite this shift, endovascular procedures like coronary angiography and PCI, which require the use of contrast media, are associated with the risk of CA-AKI [2]. Furthermore, CA-AKI appears to be linked with worse prognoses in patients undergoing PCI [3].

The percutaneous coronary intervention of chronic total occlusion (CTO PCI) is often associated with long procedural times and high contrast volumes. Femoral access is more frequently used than non-CTO PCI, requiring special devices to complete the procedure [4]. It potentially impacts renal function through device manipulations within the aorta. Long procedural times and retrograde approaches may also expose patients to a greater risk of hemodynamic instability, possibly compromising renal perfusion. Additionally, reduced left ventricular function, common in patients with CTO, may exacerbate the risk of hemodynamic instability during lengthy procedures [5]. In the literature, risk predictors of CA-AKI after coronary interventions have been primarily focused on all-comer PCI for acute and chronic coronary syndrome and rarely in dedicated CTO cohorts [6,7,8]. Furthermore, studies on the prognostic implications of CA-AKI after CTO PCI are sporadic and only short-term follow-ups exist [9,10]. In the present study, we aimed to discover risk predictors of CA-AKI and the impact of CA-AKI on one and three-year mortality in patients undergoing CTO PCI in a tertiary care center, with the scope to generate a hypothesis of whether CA-AKI is related to worse short- and long-term mortality after CTO PCI. Finally, as CA-AKI has been reported to be influenced by osmotic pressure levels and electrolytes, but without data on patients undergoing CTO PCI, we specifically analyzed them in our patient cohort [11].

## 2. Methods

### 2.1. Study Design and Patient Population

This is a single-center retrospective analysis from the prospective Vienna CTO Registry (VICTORY) in consecutive patients undergoing CTO PCI. Patients treated between 1 January 2018 and 1 October 2021 were categorized into those who developed a CA-AKI and those who did not.

All patients undergoing CTO PCI received per standard clinical practice iopamidol, a nonionic, low-osmolar contrast agent. CA-AKI was defined according to KDIGO criteria (Kidney Disease Improving Global Outcomes) as either an increase in baseline admission creatinine of ≥0.3 mg/dL within 48 h or a ≥50% increase within seven days post procedure. [12]. Patients with missing creatinine levels within seven days following the interventions were excluded. The glomerular filtration rate (GFR) was calculated using the MDRD-GFR formula [13] while plasma osmolarity was derived using baseline sodium, blood urea nitrogen (BUN), and glucose values. Osmotic pressure was estimated by multiplying plasma osmolarity by the conversion factor 19.3 [14]. Hemodynamic instability is characterized by significant deviations of the baseline blood pressure profile during the procedure. Due to the lack of documentation of blood pressure fluctuations in electronic acts, we extracted information on the periprocedural use of catecholamines or periprocedural hydration. The application of any dose of catecholamines was included in the definition of periprocedural catecholamines. Periprocedural hydration was defined as the application of more than 500 mL of intravenous fluids during the procedure. We aimed to use these parameters to count for hemodynamic fluctuations. Technical success was defined as successful stent deployment or balloon angioplasty with the attainment of TIMI 3 flow. Procedural success was defined as technical success without in-hospital major adverse cardiac and cerebrovascular events (MACCE), pericardial effusion, tamponade, or the need for urgent surgery during admission. MACCE was defined as a composite in-hospital death, myocardial infarction, and stroke during the hospital stay.

The flowchart of this study is presented in Supplementary Figure S1. Of the 271 consecutive patients in the registry, 53.5% had at least one creatinine measurement taken within seven days post-procedure and were included in the analysis. Almost half of the consecutive patients were excluded due to missing follow-up creatinine levels, which were left to the discretion of physicians at the cardiology ward. As this introduces a selection bias, this study should be considered as hypothesis-generating and its results should be validated in prospective trials. For an optimal representation of the cohort, a consecutive systematic measurement of creatinine levels post-CTO PCI should be encouraged regardless of procedural outcomes. The primary endpoint was all-cause mortality at one year, and the secondary endpoint was all-cause mortality at three years. Mortality data were retrieved from Statistics Austria’s nationwide individual patient database on 15 June 2024 and further validated using the Medical University of Vienna’s daily updated medical platform.

The study received approval from the local ethics committee (Ethics Committee of the Medical University of Vienna, EK Number 1552/2018) and was conducted in accordance with the Declaration of Helsinki.

### 2.2. Data and Statistical Analyses

Categorical variables were presented as frequencies and percentages, while continuous variables were expressed as means ± standard deviations. Baseline characteristics between patients with and without postprocedural CA-AKI were compared using the Chi-squared or Fisher’s exact test for categorical variables and Student’s *t*-test or the Mann–Whitney test for continuous variables, as appropriate. Statistical significance was defined at a two-sided alpha level of 0.05.

Correlations between baseline parameters, plasma osmotic factors, and CA-AKI were analyzed using univariable logistic regression. A multivariable logistic regression model was then constructed, incorporating significant variables from the univariable analysis along with age, sex, and contrast volume. One-year all-cause mortality was assessed using an adjusted Cox regression model that included CA-AKI, sex, age, and covariates associated with CA-AKI with a *p*-value < 0.05. Kaplan–Maier estimates were plotted for all-cause mortality at three years for patients with and without CA-AKI and investigated by the log-rank test. Additionally, a landmark analysis was performed at the one-year mark. Descriptive statistics, regression analysis, and graphical representations were performed using RStudio for Mac (v2023.09.0, PositSoftware©) with additional CRAN and Bioconductor repositories packages.

## 3. Results

### 3.1. Baseline Patient and Lesion Characteristics

Baseline patient characteristics are presented in Table 1. Of 145 patients, 36 (24.8%) developed postinterventional CA-AKI. The mean age of the cohort was 67 ± 11 years, with a predominance of male patients (75.2%). Hypertension was present in 76.6% of patients and diabetes in 33.1%, with no significant differences between those with and without CA-AKI. The incidence of chronic kidney disease, dialysis, and baseline creatinine levels were also similar between the two groups. Supplementary Table S1 summarizes plasma osmotic factors, other relevant laboratory parameters, and selected baseline medication. Both groups demonstrated baseline electrolyte levels within normal ranges, with no significant differences in electrolyte or osmotic parameters. Baseline hemoglobin was 12.9 ± 2.1 mg/dL, and median NT-proBNP was 716 pg/mL, with no significant differences between groups. However, patients with CA-AKI had higher baseline glucose levels (127 mg/dL [112;183] vs. 114 mg/dL [101;140], *p* = 0.043). Patients developing CA-AKI had fasting glucose levels predominantly corresponding to diabetes mellitus (≥126 mg/dL), significantly more frequent than in patients without CA-AKI (55.6% vs. 33.0%, *p* = 0.042). Driven by the significantly smaller number of patients with two-vessel diseases, patients with CA-AKI had numerically less multivessel disease than those without CA-AKI. (75% vs. 89%, *p* = 0.073). Univariable logistic regression revealed no significant correlations between electrolyte levels, osmotic pressure, or baseline glucose levels and the occurrence of CA-AKI (OR 1.01, CI [1.00;1.02], *p* = 0.07).

Lesion characteristics are detailed in Table 2. Lesion characteristics were similar across both groups. Patients had comparable lesion complexity, with a median J-CTO score of 2. A moderate to severe calcification in the CTO lesion was observed in 29% of patients, and long lesions (>20 mm) were present in 62.8% of cases.

### 3.2. Procedural Characteristics and Outcomes

Procedural characteristics and outcomes are presented in Table 3. The most common vascular access was bifemoral (48.3%), followed by biradial (31.7%), with no significant differences between groups. The use of the retrograde approach was similar between the groups, as were the stent sizes in length and diameter. Total procedural and fluoroscopy times were numerically higher in patients who developed CA-AKI, with a significantly higher radiation dose in the CA-AKI group (22087 vs. 13326 µGy·cm^2^, *p* = 0.041), reflecting the longer procedural and fluoroscopic times. Technical and procedural success rates were comparable between groups. The MACCE rate was 4.1%, with a higher, though non-significant, incidence in patients with CA-AKI (8.3% vs. 2.75%, *p* = 0.162). Periprocedural catecholamine administration and hydration were similar between groups. Patients with CA-AKI had higher post-interventional creatinine levels (1.02 mg/dL [0.84;1.30] vs. 1.73 mg/dL [1.45;2.45], *p* < 0.001).

Univariable logistic regression showed an unadjusted correlation of total DAP in mGy·cm^2^, (OR 1.044 CI [1.01;1.08], *p* = 0.011) with CA-AKI. (Supplementary Table S2) Each 1000 µGy·cm^2^ increase in total DAP is associated with 4.4% increase in risk for CA-AKI. In multivariable logistic regression, total DAP remained an independent predictor of CA-AKI. With 1000 µGy·cm^2^ increase in total DAP there is an adjusted risk increase of 5.6% for CA-AKI (*p* = 0.006).

### 3.3. All-Cause Mortality

**Figure 1:**
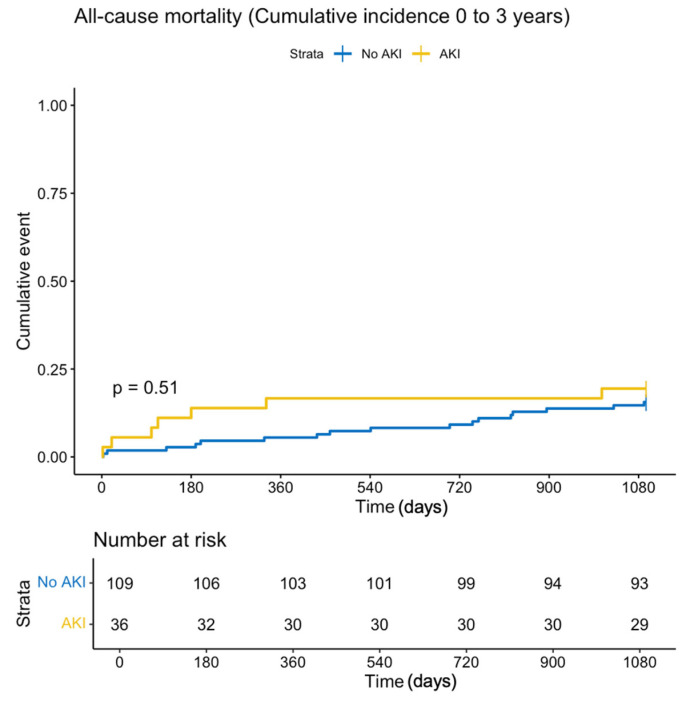
Kaplan–Maier estimates for all-cause mortality at three-year follow-up.

One-year all-cause mortality was 16.7% in the CA-AKI group compared to 5.5% in the non-CA-AKI group (log-rank *p* = 0.032, HR 3.23, CI [1.04–10.02], *p* = 0.042) (Supplementary Figure S2). In multivariable Cox regression analysis, which included CA-AKI, sex, age, and total DAP, CA-AKI was an independent predictor of one-year mortality (adjusted HR 5.3, CI [1.52–18.51], *p* = 0.009). Patient age also independently predicted one-year survival (adjusted HR 1.07, CI [1.01–1.14], *p* = 0.022). The Mehran risk score did not significantly correlate with one-year mortality in this cohort (OR 1.09, CI [0.98–1.21], *p* = 0.096). All-cause mortality at three years did not show significant differences between groups. (Figure 1)

**Figure 2:**
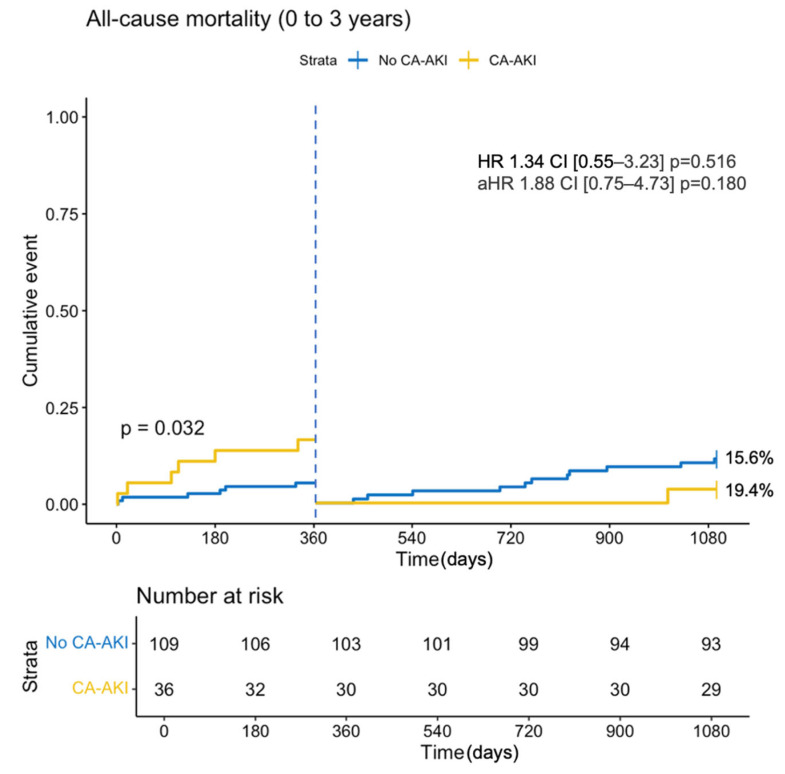
Kaplan–Meier estimates of the three-year survival in patients with CA-AKI (AKI) and those without (no AKI) with one-year landmark. One-year mortality log-rank *p* = 0.032.

A landmark analysis at the one-year mark was performed (Figure 2). The significance of a higher all-cause mortality rate at one year in the CA-AKI group diminished over the longer three-year observation period (Supplementary Table S3).

## 4. Discussion

In this retrospective analysis of a prospective CTO registry, the main findings were as follows. (1) Baseline renal function and electrolyte levels did not significantly differ between patients with and without CA-AKI. (2) No significant differences in lesion characteristics were observed between the two study groups. (3) Patients developing CA-AKI underwent longer procedures with the need for higher contrast volumes reflected by significantly higher radiation doses. (4) CA-AKI was an independent predictor for all-cause mortality at one year but not at three years.

Despite meticulous procedural planning for PCI, we observed a high rate of CA-AKI. The rate of CA-AKI was higher than in other all-comer PCI cohorts, similar to CTO PCI studies [7,10,15]. Interestingly, neither baseline creatinine levels, electrolytes, plasma osmolarity (as a marker of hydration status), nor the therapy with diuretics were predictive for CA-AKI. In contrast to patients not developing CA-AKI, those with CA-AKI had mainly fasting glucose levels of ≥126 mg/dL—suggesting that uncontrolled diabetes mellitus at the intervention timepoint could jeopardize the kidney. In line with this, diabetes mellitus was among other clinical and procedural factors incorporated in the Mehran score for predicting the risk of CA-AKI after PCI [6]. Thus, clinical aspects and metabolic abnormalities beyond the baseline kidney function or volume status may have contributed to the CA-AKI risk. Although electrolyte abnormalities were reported to be linked to worse cardiovascular outcomes and an increased risk of CA-AKI; these effects seem to be less prominent in CTO-PCI patients [11,16,17].

In an all-comer PCI registry, Azzalini et al. highlighted the influence of hemodynamic parameters over baseline creatinine on the development of CA-AKI. After adjustment, they reported several predictive variables for CA-AKI, including hypotension during or before the PCI, acute coronary syndrome, reduced left ventricular ejection fraction, and reduced hemoglobin levels, followed by age and contrast media amount [7]. However, with balanced catecholamine administration and periprocedural hydration between our groups, these hemodynamic risk factors could have only played a minor role in our cohort, as CTO PCI is mainly performed in stable patients.

Besides these clinical features, lesions and procedural complexity likely influence the risk of developing CA-AKI. Mankerious et al. demonstrated that the Target Vessel Syntax Score predicted both in-hospital outcomes and CA-AKI in all-comers undergoing rotational atherectomy [18,19]. In a dedicated CTO-PCI registry, calcification, use of rotational atherectomy, and the J-CTO score were significantly more common in the CA-AKI population but were not predictive for the development of CA-AKI [10]. Lesion complexity and J-CTO score failed to predict CA-AKI in our cohort. Instead, procedural effort—reflected by longer procedure times, prolonged fluoroscopy duration, and larger contrast volumes—was more pronounced in patients who developed CA-AKI. Multivessel disease was numerically lower in CA-AKI patients, driven by significantly fewer instances of two-vessel disease. A multivessel disease may indicate a history of cardiovascular care or a longer regimen of optimized medical therapy. Some cardiovascular medications, such as statins or thrombocyte aggregation inhibitors, may stabilize peripheral atherosclerotic plaques and introduce kidney protection during interventions [20,21].

In the study by Azzalini et al., hemodynamic parameters and the amount of contrast media had a moderate influence but were still significant predictors of CA-AKI (adjusted odds ratio of 1.18 per 100 mL of contrast used) [7]. If not excessively administered, contrast media could be well tolerated in PCI. Although our patients received higher contrast volumes compared to all-comer PCI, as commonly observed in CTO PCI [10], no significant correlation with CA-AKI was observed. However, patients developing CA-AKI underwent longer procedures requiring higher contrast volumes with prolonged procedural effort, reflected by significantly higher radiation doses.

CA-AKI following CTO PCI emerged as an independent predictor for one-year all-cause mortality. However, it remains unclear whether this increased mortality risk resulted from long-term deterioration in kidney function or from the same factors predisposing patients to CA-AKI also driving mortality. CTO PCI is a complex procedure with high complication rates in highly comorbid patients [22], and elevated mortality could be attributed to baseline and procedural risk. These patients are additionally affected by increased MACCE rate, characterized by an eventful postinterventional timeline with a gradual increase in cardiovascular mortality risk [22,23,24]. Accounting for these confounders, kidney injury may be an indicator and not directly influence the mortality risk.

In line with our study, CA-AKI predicted one-year all-cause mortality in a large, contemporary German CTO registry [10]. Conversely, the predictive value for mortality at two years could not be proven in another international multicenter study [9]. In our study, CA-AKI lost its initial predictive value for all-cause mortality over the extended three-year observation period. We hypothesize that the initial mortality risk at one year in CA-AKI patients is likely driven by persistent renal injury. In contrast, kidney injury may reverse in many CA-AKI patients in the longer term. Indeed, only a tiny proportion of patients with CA-AKI after coronary angiography experience persistent renal damage, while others normalize their creatinine levels [25]. The long-term course of renal recovery in patients with CA-AKI following CTO PCI is poorly understood, as dedicated longitudinal studies on renal function are lacking. This highlights the value of prospective studies on the temporal course of renal function after CTO PCI and PCI in general. To appropriately objectify renal deterioration, one should comprehensively assess not only creatinine level abnormalities but also other parameters such as albuminuria or hyperuricemia. Long-term monitoring and optimized communication between treating interventionalists and medical caregivers need to be established to enhance patient care after CTO PCI and identify patients with persistent damage. Additionally, future studies are needed to better understand the impact of renal function after PCI on long-term outcomes. On another note, as a relation between procedural characteristics and renal injury is reasonable, a systematic baseline risk stratification facilitated by cardiac structural and functional assessment and tissue characterization should be encouraged to ensure uncomplicated interventions [26,27]. Based on our observations, we advocate for careful patient selection and risk-benefit assessment prior to complex interventions such as CTO PCI.

### Limitations

Firstly, as a single-center retrospective analysis, this study has inherent limitations, and the results should be considered hypothesis-generating rather than definitive. Secondly, while post-procedure lab tests were performed per standard protocol, the serial monitoring of creatinine levels was left to the discretion of the treating physicians in the cardiology ward. Consequently, patients without available postprocedural creatinine levels were excluded from the analysis, which may have introduced selection bias. Specifically, physicians may have opted not to conduct post-interventional lab tests in patients with fewer comorbidities or an uneventful clinical course, potentially inflating the incidence of CA-AKI in our study. Thirdly, we did not extract information on hemodynamics during the procedure. However, we included several procedure-related variables such as procedural time, fluoroscopy time, lesion complexity parameters, administration of catecholamines and periprocedural hydration, all of which would be indicative of risk for peri-procedural events. Fourthly, individual complications during the procedure may have influenced the development of CA-AKI. Still, as this parameter is not available upfront, it has not been added to our risk prediction model. Lastly, prophylactic hydration was at the discretion of the treating physicians, and data on hydration protocols were not retrievable from patient records.

## 5. Conclusions

In this retrospective analysis, CA-AKI was an independent predictor of all-cause mortality one year after CTO PCI. This enhanced mortality risk did not persist over three years. Procedural effort, marked by higher radiation doses, contributed to the risk of developing CA-AKI. These findings underscore the need to better identify patients at risk for CA-AKI and understand the underlying mechanisms. Further dedicated, prospective studies are needed to validate these hypotheses.

## Figures and Tables

**Table 1:** Baseline characteristics of the study cohort.

	Total *n* = 145	No CA-AKI *n* = 109	CA-AKI *n* = 36	*p* Value
Male sex	109 (75.2%)	84 (77.1%)	25 (69.4%)	0.487
Age (y)	67.3 (11.5)	67.3 (11.2)	67.3 (12.4)	0.996
BMI	26.9 [24.7;31.1]	26.8 [24.5;30.8]	28.5 [25.2;32.8]	0.217
Diabetes	48 (33.1%)	35 (32.1%)	13 (36.1%)	0.812
Dyslipidemia	107 (73.8%)	83 (76.1%)	24 (66.7%)	0.367
Hypertension	111 (76.6%)	85 (78.0%)	26 (72.2%)	0.631
Current smoker	38 (26.2%)	27 (24.8%)	11 (30.6%)	0.641
Prior MI	62 (42.8%)	48 (44.0%)	14 (38.9%)	0.729
Prior PCI	81 (55.9%)	65 (59.6%)	16 (44.4%)	0.162
Prior CABG	20 (13.8%)	13 (11.9%)	7 (19.4%)	0.272
PAD	31 (21.4%)	23 (21.1%)	8 (22.2%)	1.000
TIA/Stroke	5 (3.45%)	4 (3.67%)	1 (2.78%)	1.000
History of Heart Failure	27 (18.6%)	19 (17.4%)	8 (22.2%)	0.694
Chronic pulmonary disease ^§^	20 (13.8%)	15 (13.8%)	5 (13.9%)	1.000
Chronic kidney disease *	54 (37.2%)	38 (34.9%)	16 (44.4%)	0.405
Dialysis	1 (0.93%)	1 (1.16%)	0 (0.00%)	1.000
Baseline creatinine (mg/dL)	1.04 [0.87;1.31]	1.02 [0.87;1.31]	1.09 [0.87;1.33]	0.589
GFR (mL/min/1.73 m^2^)	71.0 [53.1;87.6]	71.5 [55.0;87.5]	65.6 [50.9;90.9]	0.640
Fasting glucose				
100–125 mg/dL	59 (40.7%)	47 (43.1%)	12 (33.3%)	0.042
≥126 mg/dL	56 (38.6%)	36 (33.0%)	20 (55.6%)	

* GFR < 60mL/min/1.73m^2^, ^§^ defined as a composite of chronic obstructive pulmonary disease; asthma; pulmonary fibrosis; any condition requiring chronic use of bronchodilators; BMI—body mass index; MI—myocardial infarction; PCI—percutaneous coronary intervention; CABG—coronary artery bypass graft; PAD—peripheral artery disease; TIA—transient ischemic attack; and GFR—glomerular filtration rate.

**Table 2:** Lesion characteristics.

	Total *n* = 145	No CA-AKI *n* = 109	CA-AKI *n* = 36	*p* Value
Number of diseased vessels				0.008
1	21 (14.5%)	12 (11.0%)	9 (25.0%)	
2	53 (36.6%)	47 (43.1%)	6 (16.7%)	
3	71 (49.0%)	50 (45.9%)	21 (58.3%)	
Multivessel disease	124 (85.5%)	97 (89.0%)	27 (75.0%)	0.073
CTO vessel				0.186
LAD	37 (25.5%)	25 (22.9%)	12 (33.3%)	
CX	25 (17.2%)	22 (20.2%)	3 (8.33%)	
RCA	83 (57.2%)	62 (56.9%)	21 (58.3%)	
J-CTO score	2 [1;3]	2 [1;3]	2 [1;3]	0.574
In-stent CTO	13 (8.97%)	10 (9.17%)	3 (8.33%)	1.000
CTO involving a bifurcation Lesion	29 (20.0%)	25 (22.9%)	4 (11.1%)	0.194
Calcification (moderate–severe)	42 (29.0%)	31 (28.4%)	11 (30.6%)	0.976
Length > 20 mm	91 (62.8%)	67 (61.5%)	24 (66.7%)	0.718
Proximal cap ambiguity	52 (35.9%)	42 (38.5%)	10 (27.8%)	0.334
Absence of interventional collaterals	14 (9.66%)	10 (9.17%)	4 (11.1%)	0.749
Moderate–severe tortuosity	17 (11.7%)	12 (11.0%)	5 (13.9%)	0.765
Circumflex CTO	28 (19.3%)	22 (20.2%)	6 (16.7%)	0.826
Ostial CTO	20 (13.8%)	14 (12.8%)	6 (16.7%)	0.582
Aorto-ostial CTO	5 (3.45%)	3 (2.75%)	2 (5.56%)	0.598

CTO—chronic total occlusion; LAD—left anterior descending; CX—circumflex coronary artery; and RCA—right coronary artery.

**Table 3:** Procedural characteristics and in-hospital outcomes.

	Total *n* = 145	No CA-AKI *n* = 109	CA-AKI *n* = 36	*p* Value
Use of radial access				0.183
Biradial	46 (31.7%)	39 (35.8%)	7 (19.4%)	
Radial and femoral	29 (20.0%)	20 (18.3%)	9 (25.0%)	
CTO final strategy				0.881
Antegrade	120 (82.8%)	91 (83.5%)	29 (80.6%)	
Retrograde	25 (17.2%)	18 (16.5%)	7 (19.4%)	
Rotational atherectomy	9 (6.21%)	6 (5.50%)	3 (8.33%)	0.690
Number of stents	2 [1;3]	2 [1;3]	2 [1;2]	0.759
Maximal stent size (mm)	3.27 (0.56)	3.24 (0.58)	3.36 (0.48)	0.271
Total stent length (mm)	54.5 [32.2;80.0]	56.0 [33.0;81.2]	50.5 [30.5;75.5]	0.395
Mehran risk score	11.3 (5.52)	11.2 (5.42)	11.6 (5.85)	0.674
Total procedural time (min)	170 [135;210]	165 [127;205]	180 [153;211]	0.103
Contrast volume (mL)	250 [178;330]	240 [170;330]	280 [214;338]	0.173
Total DAP (µGy·m^2^)	14,147 [7815;24,528]	13,326 [7292;22,090]	22,087 [9313;29,823]	0.041
Periprocedural hydration	62 (42.8%)	47 (43.1%)	15 (41.7%)	1.000
Periprocedural catecholamines	20 (13.8%)	15 (13.8%)	5 (13.9%)	1.000
Fluoroscopy duration (min)	46.3 [28.0;65.4]	43.0 [26.1;59.5]	52.1 [35.6;73.1]	0.138
Technical success	115 (79.3%)	86 (78.9%)	29 (80.6%)	1.000
Procedural success	105 (72.4%)	80 (73.4%)	25 (69.4%)	0.807
In-hospital MACCE	6 (4.14%)	3 (2.75%)	3 (8.33%)	0.162
Post max creatinine (mg/dL)	1.16 [0.88;1.63]	1.02 [0.84;1.30]	1.73 [1.45;2.45]	<0.001

CTO—chronic total occlusion; DAP—dose area product; and MACCE—major adverse cardiovascular and cerebrovascular events.

## Data Availability

Data could be shared by the principal investigator upon reasonable request.

## Supplementary Materials

The following supporting information can be downloaded at: https://www.mdpi.com/article/10.3390/jcm13247676/s1, Figure S1: Flowchart of the study; Figure S2: Kaplan Maier estimates for all-cause mortality at one-year follow-up; Table S1: Baseline laboratory, osmotic parameters, and medication at admission; Table S2: Univariable and multivariable logistic regression analysis for predictors of CA-AKI; Table S3: Univariable and multivariable cox-regression and log-rank test for all-cause mortality in one- and three-years follow-up.
