# Supplementary material for: Postprocedural Contrast-Associated Acute Kidney Injury and Prognosis of Patients Undergoing Recanalization of Chronic Total Occlusions
Source: J Clin Med. 2024 Dec 16;13(24):7676. doi: 10.3390/jcm13247676 (PMC11677092; doi:10.3390/jcm13247676)
Supplement: Supplementary file 1 [file jcm-13-07676-s001.zip › jcm-3348743-supplementary.pdf]

Supplementary Materials

Supplementary Figure S1. Flowchart of the study

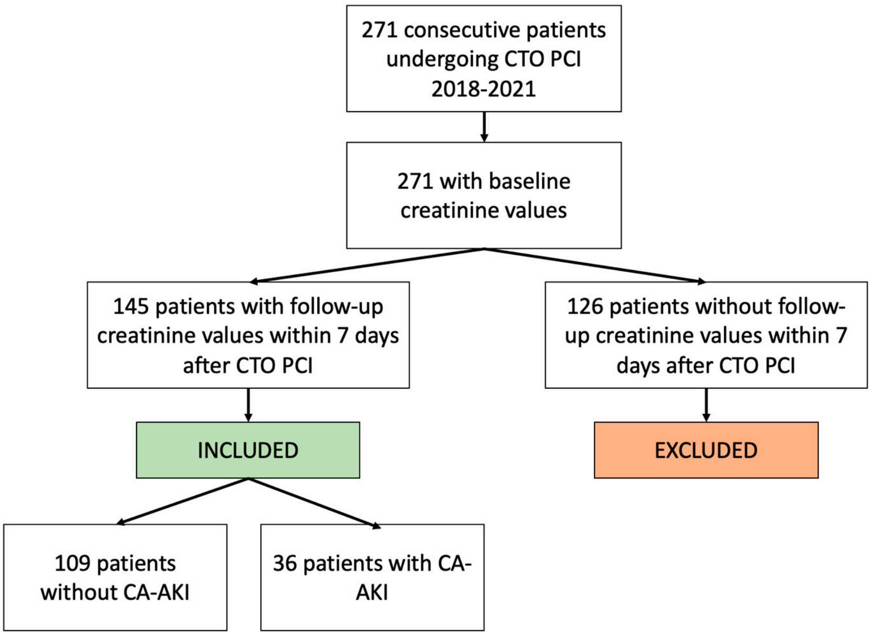

CTO – chronic total occlusion, PCI – percutaneous coronary intervention, CA-AKI contrast-associated acute kidney injury

Supplementary Table S1. Baseline laboratory, osmotic parameters, and medication at admission.

	Total **N=145**	No CA-AKI **N=109**	CA-AKI **N=36**	**p Value**
Osmotic pressure (mmHg)	5597 [5497;5682]	5594 [5484;5684]	5599 [5529;5668]	0.816
**Osmolarity (mOsm/l)**	288 [97.0;293]	287 [35.7;293]	290 [284;293]	0.336
**Baseline Hb (g/dL)**	12.9 (2.08)	12.9 (2.02)	12.7 (2.28)	0.635
**LDL (mg/dl)**	65.8 [48.0;84.0]	66.1 [50.5;83.8]	64.6 [45.8;85.0]	0.576
**NT-proBNP (pg/ml)**	716 [228;2066]	626 [227;1652]	1006 [376;4978]	0.124
**Sodium (mmol/l)**	139 [137;141]	139 [137;141]	138 [136;140]	0.087
**Potassium (mmol/l)**	4.18 (0.54)	4.17 (0.54)	4.22 (0.57)	0.657
**Calcium (mmol/l)**	2.26 [2.17;2.36]	2.26 [2.18;2.36]	2.26 [2.10;2.34]	0.287
**Magnesium (mmol/l)**	0.83 [0.76;0.89]	0.82 [0.76;0.89]	0.84 [0.78;0.96]	0.251
**Blood urea nitrogen (mg/dl)**	17.4 [13.1;24.3]	17.3 [13.6;23.8]	18.2 [13.1;29.9]	0.570
**Albumin (g/dl)**	39.8 [36.8;42.9]	40.2 [36.8;43.2]	39.0 [36.8;42.3]	0.405
**Glucose (mg/dl)**	117 [103;146]	114 [101;140]	127 [112;183]	0.043
**ACE-Inhibitors / ARBs**	103 (73.0%)	80 (74.8%)	23 (67.6%)	0.553
**Beta-Blocker**	123 (87.2%)	94 (87.9%)	29 (85.3%)	0.769
**Ca-Antagonist**	21 (14.9%)	15 (14.0%)	6 (17.6%)	0.809
**Diuretics**	73 (51.8%)	52 (48.6%)	21 (61.8%)	0.254

**Aspirin**	133 (94.3%)	101 (94.4%)	32 (94.1%)	1.000
**Statins**	114 (80.9%)	87 (81.3%)	27 (79.4%)	1.000

LDL – low density lipoprotein, NT-proBNP - N-terminal pro–B-type natriuretic peptide.

Supplementary Table S2. Univariable and multivariable logistic regression analysis for predictors of CA-AKI.

Parameter	Univariable OR [CI]	p value	Multivariable OR [CI]	p value
Male sex	0.68 [0.29;1.61]	0.369	0.461 [0.17, 1.26]	0.132
Age (y)	1.00 [0.96;1.03]	0.824	1.006 [0.97, 1.05]	0.780
BMI	1.03 [0.98;1.09]	0.207		
Diabetes	1.20 [0.53;2.63]	0.659		
Dyslipidemia	0.63 [0.28;1.46]	0.274		
Hypertension	0.73 [0.31;1.80]	0.485		
Current smoker	1.34 [0.56;3.06]	0.498		
Prior MI	0.81 [0.37;1.75]	0.598		
Prior PCI	0.54 [0.25;1.17]	0.119		
Prior CABG	1.79 [0.61;4.86]	0.276		
PAD	1.08 [0.41;2.62]	0.874		
TIA/ Stroke	0.83 [0.03;6.21]	0.871		
Heart Failure	1.36 [0.51;3.39]	0.525		
Chronic Pulmonary Disease	1.03 [0.31;2.94]	0.960		
CKD	1.71 [0.75;3.84]	0.200		
ACE-Inhibitors /ARBs	0.70 [0.31;1.69]	0.423		
beta-Blocker	0.79 [0.27;2.70]	0.688		
Ca-Antagonist	1.33 [0.43;3.65]	0.603		
Diuretics	1.70 [0.77;3.83]	0.188		
Baseline creatinine (mg/dl)	1.30 [0.85;1.98]	0.230		
Baseline Hb (g/dL)	0.95 [0.80;1.14]	0.610		
LDL (mg/dl)	1.00 [0.99;1.01]	0.786		
NT-proBNP (pg/ml)	1.00 [1.00;1.00]	0.054		
Post maximal creatinine (mg/dl)	7.88 [3.39;18.3]	<0.001		
GFR (ml/min/1.73m2)	1.00 [0.99;1.01]	0.963		
CTO Length (mm)	1.00 [0.98;1.02]	0.902		
J-CTO score	0.87 [0.63;1.20]	0.399		
Total procedural time (min)	1.00 [1.00;1.01]	0.224		
Contrast volume (ml)	1.00 [1.00;1.01]	0.254	1.001 [0.99, 1.01]	0.712
Fluoroscopy duration (min)	1.01 [1.00;1.02]	0.149		
In-stent CTO	0.93 [0.19;3.32]	0.917		
CTO involving a bifurcation lesion	0.43 [0.12;1.24]	0.125		
Calcification (moderate-severe)	1.11 [0.47;2.51]	0.802		
Length >20 mm	1.25 [0.57;2.84]	0.588		

Proximal cap ambiguity	0.62 [0.26;1.39]	0.251		
Absence of interventional collaterals	1.26 [0.32;4.14]	0.720		
Moderate-severe tortuosity	1.32 [0.39;3.93]	0.637		
Circumflex CTO	0.80 [0.27;2.09]	0.667		
Ostial CTO	1.37 [0.44;3.79]	0.565		
Aorto-ostial CTO	2.11 [0.24;14.4]	0.461		
Main vascular access:				
Biradial	0.46 [0.16;1.16]	0.100		
Radial and femoral	1.13 [0.42;2.89]	0.803		
Retrograde	1.23 [0.44;3.17]	0.680		
Rotational atherectomy	1.59 [0.30;6.61]	0.550		
Technical success	1.09 [0.44;3.04]	0.853		
Procedural success	0.82 [0.36;1.94]	0.645		
MACCE	3.18 [0.52;19.3]	0.196		
Hemoglobin	0.95 [0.79;1.13]	0.545		
Sodium (mmol/l)	0.91 [0.81;1.03]	0.154		
Potassium (mmol/l)	1.19 [0.58;2.44]	0.643		
Calcium (mmol/l)	0.15 [0.01;1.75]	0.129		
Magnesium (mmol/l)	8.90 [0.38;206]	0.173		
Blood urea nitrogen (mg/dl)	1.01 [0.98;1.04]	0.497		
Osmotic pressure (mmHg)	1.00 [1.00;1.00]	0.365		
Osmolarity (mOsm/l)	1.00 [1.00;1.01]	0.186		
Albumin (g/dl)	0.96 [0.89;1.03]	0.226		
Glucose (mg/dl)	1.01 [1.00;1.02]	0.070		
LDL-Cholesterol (mg/dl)	1.00 [0.98;1.01]	0.612		
HbA1C (%)	0.89 [0.58;1.36]	0.587		
Mehran risk score	1.02 [0.95;1.09]	0.658		
Total DAP (per 1000 μGy·cm^2)	1.04 [1.01;1.08]	**0.011**	1.056 [1.02, 1.10]	0.006
Multivessel	0.37 [0.14;1.01]	0.053		
Periprocedural hydration	0.94 [0.43;2.03]	0.885		
Periprocedural cathecolamines	1.03 [0.31;2.94]	0.960		
Statins	0.88 [0.34;2.47]	0.795		
Aspirin	0.91 [0.19;7.14]	0.914		

Supplementary Table S3. Univariable and multivariable cox-regression and log-rank test for all-cause mortality in one- and three-years follow-up.

	**No CA-AKI (n=109)**	**CA-AKI (n=36)**	**p value log-rank**	**HR (95% CI)**	**p value**	**aHR (95% CI)**	**p value**
**One year mortality**	6 (5.50%)	6 (16.7%)	0.032	3.232 [1.04-10.02]	0.042	5.306 [1.52-18.5]	0.009
**Three year-mortality**	17 (15.6%)	7 (19.4%)	0.51	1.338 [0.55-3.22]	0.516	1.881 [0.74-4.73]	0.180

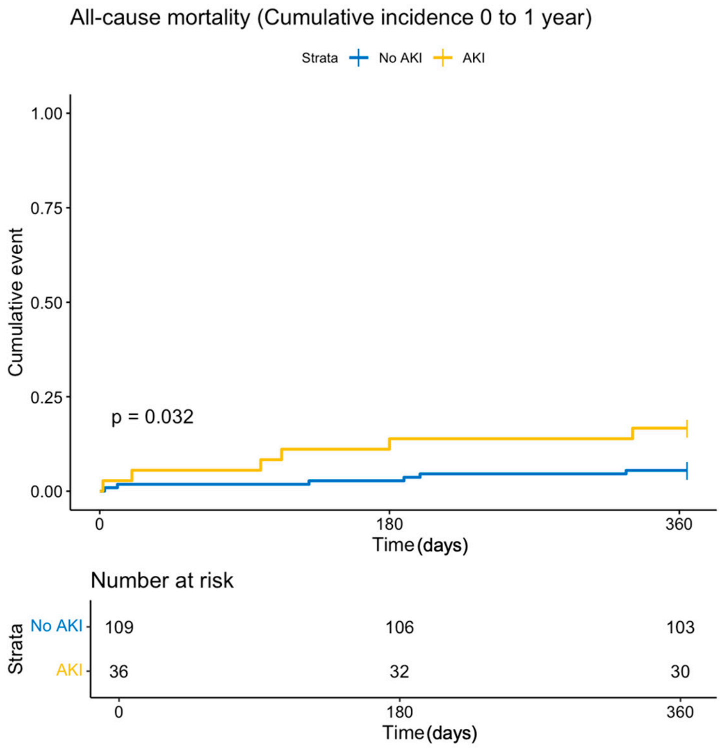

Supplementary Figure S2. Kaplan Maier estimates for all-cause mortality at one-year follow-up.
